# GLPp16 gene amplification induces susceptibility to high-grade urothelial carcinoma

**DOI:** 10.3389/fonc.2024.1495381

**Published:** 2024-11-26

**Authors:** Yuxin Liu, Qihao Sun, Houtao Long, Daofeng Zhang, Junhao Zheng, Haiyang Zhang

**Affiliations:** ^1^ Department of Urology, Shandong Provincial Hospital, Shandong University, Jinan, China; ^2^ Department of Urology, Shandong Provincial Hospital Affiliated to Shandong First Medical University, Jinan, China; ^3^ Knuppe Molecular Urology Laboratory, Department of Urology, School of Medicine, University of California, San Francisco, San Francisco, CA, United States

**Keywords:** FISH, GLPp16, nomogram, pathological grading, urothelial carcinoma

## Abstract

**Background:**

Urothelial carcinoma is a common malignant tumor of the urinary system, with prognosis linked to pathological grade and TNM stage. Alterations in chromosomes 3, 7, and 17, along with the P16 locus on chromosome 9 (CSP3, CSP7, CSP17, and GLPp16), are associated with cancer progression and may serve as important biomarkers. This study aimed to explore the relationships between these chromosomal factors and the pathological grade and TNM stage of UCC, potentially leading to a novel diagnostic approach that enhances patient stratification and treatment planning.

**Methods:**

A retrospective analysis was conducted on 149 patients to evaluate the correlation between CSP3, CSP7, CSP17, GLPp16, TNM stage, and pathological grade using chi-square tests and logistic regression. Immunohistochemistry was employed to assess the associated changes.

**Results:**

Univariate analysis indicated that only CSP7 and GLPp16 were significantly associated with pathological grade. Logistic regression linked GLPp16 and gender to pathological grade in urothelial carcinoma. A nomogram model incorporating these factors demonstrated reliable calibration in the training set (non-significant Hosmer-Lemeshow test, P = 0.436; AUC = 0.785, 95% CI: 0.707 - 0.863) and effective discrimination in the test set (AUC = 0.740, 95% CI: 0.559 - 0.920). Immunohistochemistry revealed P16 gene deletion in low-grade urothelial carcinoma and amplification in high-grade urothelial carcinoma.

**Conclusion:**

Mutations at the GLPp16 were significantly correlated with the pathological grade of urothelial carcinoma. Additionally, the amplification of GLPp16 was recognized as a contributing factor to the development of high-grade urothelial carcinoma.

## 1 Introduction

Bladder cancer is the most prevalent type of urinary tract cancer, ranking as the 10th most common cancer globally (1). The incidence rate of urothelial carcinoma is higher in males, with approximately 5% of males affected, making it the sixth most common cancer among men (2). Urothelial carcinoma represents over 90% of bladder cancer cases (3). Pathological grade and TNM stage are recognized as pivotal factors influencing the prognosis of urothelial carcinoma (4). Patients with higher grade and TNM stage face an elevated risk of disease-specific mortality, indicating prolonged treatment duration (5). Thus, early detection and accurate pathological staging and grading play a crucial role in guiding the treatment and prognosis of urothelial carcinoma (6).

Currently, the standard method for diagnosing urothelial carcinoma is through pathological biopsy (4), often requiring invasive cystoscopy and ureteroscopy to obtain tissue samples, posing challenges for early or *in situ* urothelial carcinoma detection (7). Moreover, cystoscopy may not be suitable for all patients. Hence, there is a pressing need for a noninvasive and dependable diagnostic approach to aid in diagnosing, staging, and grading urothelial carcinoma (8). Urine-exfoliated cells are commonly utilized in clinical settings due to their simplicity, noninvasiveness, and cost-effectiveness, despite their limitations of low sensitivity and difficulty in detecting low-grade tumors (9). Fluorescence *in situ* hybridization (FISH) of urine-exfoliated cells is a noninvasive urine-based test currently applied to detect prevalent genetic alterations associated with bladder cancer, facilitating diagnosis and monitoring (10). Previous studies have demonstrated that a composite FISH detection of aneuploidy at chromosome 3, 7, 17, and chromosome 9 P16 gene loci (CSP3, CSP7, CSP17, and GLPp16) can accurately identify urothelial carcinoma cells from exfoliated urine cells with high sensitivity and specificity (11, 12). Hence, our study aimed to investigate the correlation between chromosomes 3, 7, 17, and the P16 gene locus on chromosome 9 with the pathological stage and grade of urothelial carcinoma, providing a new supplementary method for the clinical diagnosis of urothelial carcinoma.

## 2 Methods

### 2.1 Study design and patients

Data was collected from patients diagnosed with urothelial carcinoma at Shandong Provincial Hospital who received treatments from January 2022 to December 2023. Based on the patients’ clinical conditions, physical status, and individual needs, our center provided a range of treatment options, including transurethral resection of bladder tumor (TURBT), partial cystectomy, radical cystectomy, nephroureterectomy, and chemotherapy. The inclusion criteria encompassed patients meeting the following conditions: (1) a pathological diagnosis of urothelial carcinoma, and (2) positive FISH test results. A total of 149 patients meeting these dual criteria were included in the study. Among them, data from 119 patients treated during the first 20 months were allocated to the training set, while the remaining 30 patients were assigned to the test set.

### 2.2 Collection and processing of specimens

200 ml of morning urine was collected with stringent measures to prevent contamination during sample collection. Samples were promptly sent for testing to ensure their integrity. Urine collection was conducted prior to any procedures that could potentially impact test outcomes, including enhanced CT or MRI scans, cystoscopy, ureteroscopy, prostate rectal examination, or catheterization.

### 2.3 FISH

The urine samples were processed into cytospin slides for analysis as exfoliated cell specimens. Following enzymatic digestion and ethanol dehydration, the cytospin slides underwent CSP3/CSP7 and CSP17/GLPp16 fluorescent probe hybridizations, washings, restainings, and observation of FISH staining results.

Urine samples were collected from 20 healthy volunteers. The FISH experiments were performed as previously described, with 100 clearly defined signals observed for each probe combination. The percentages of cells exhibiting various types of abnormalities were calculated, and a threshold was established, defined as the mean plus three times the standard deviation (M + 3SD). The interpretation of the FISH results required analyzing 100 non-overlapping urothelial cells for each probe in each sample. This analysis involved calculating the percentage of cells with different abnormal conditions. Results exceeding the threshold were designated as positive, while those below the threshold were classified as negative.

### 2.4 Pathological detection

All pathologies were surgically removed or extracted during cystoscopy by the chief urology physician at Shandong Provincial Hospital. Subsequently, they were reviewed and diagnosed by the senior doctor of the pathology department at the same hospital.

### 2.5 Model construction and validation

For the patients in the training set, a univariate analysis was conducted to examine the relationship between chromosome mutations and pathological grading. Relevant chromosomal variations, age, and gender were jointly analyzed through a multifactor analysis, gradually eliminating non-significant factors and retaining influential factors with *p <* 0.05 to establish a logistic regression model. Utilizing the decision curve analysis (DCA) to calculate the net benefit within the threshold probability range. Establishment of a calibration curve and performing the Hosmer-Lemeshow test to assess the model’s fit. Performance evaluation of the model in the training and test sets using the receiver operating characteristic (ROC) curve.

### 2.6 Immunohistochemistry

Freshly collected urothelial carcinoma tissues were initially fixed in 4% paraformaldehyde for 3 hours. Subsequently, the tissues underwent a series of dehydration processes: immersion in 75% ethanol for 1.5 hours, 95% ethanol for 1.5 hours, 95% ethanol for 1 hour, anhydrous ethanol for 1.5 hours, anhydrous ethanol for 1 hour, and xylene I and xylene II for 0.5 hours each. After dehydration, the tissues were embedded in paraffin and sectioned into 4-micrometer slices. The slices were subjected to high-temperature antigen retrieval at 65°C for 20 minutes, followed by deparaffinization in xylene for 1 to 3 minutes and rehydration through an ethanol gradient. Microwave antigen retrieval was then performed, and endogenous peroxidase and avidin activities were blocked using an endogenous peroxidase blocking buffer (purchased from Dowobio, China, DW 2176). Afterward, the cells were blocked with normal goat serum (purchased from ZSGB-BIO, China, ZLI-9022) for 30 minutes at room temperature. Following the blocking step, the cells were incubated with the primary antibody against p19, (dilution multiple: 1:200) (purchased from CST, USA, #18769) at 4°C for 16 hours. Post incubation, the cells were exposed to mouse horseradish peroxidase solution at room temperature for 120 minutes and stained with 3,3’-diaminobenzidine (purchased from Beyotime Biotechnology, China, P0202) for 5 minutes. After counterstaining with hematoxylin for 2 minutes, the excess dye was removed with PBS washing, and differentiation was performed in 1% hydrochloric acid alcohol for 3 seconds. Finally, the cells underwent dehydration in 70% ethanol for 1 minute, 80% ethanol for 1 minute, 95% ethanol for 2 minutes, anhydrous ethanol for 4 minutes, xylene I for 3 minutes, and xylene II for 3 minutes before the sections were sealed. The results were observed with the optical microscope. Three high-power fields were assessed for each section, and each measurement was conducted in triplicate.

### 2.7 Statistical analysis

The collected data were statistically analyzed using SPSS 20.0 software. Chi-square tests were utilized to assess the relationship between FISH detection results and the TNM staging and pathological grading of urothelial carcinoma. Multivariate analysis using R packages was employed to examine the correlation between multiple factors and the pathological grading of urothelial carcinoma, followed by binary Logistic regression analysis. A significance level of *p <* 0.05 was used to determine statistical significance.

## 3 Results

### 3.1 Patient cohort

The basic information of all patients is shown in **Table 1**. In the training set, there were 119 patients with urothelial carcinoma, consisting of 101 males (84.87%) and 18 females (15.13%), with a mean age of 66.52 ± 12.62 years. According to the TNM staging classification, there were 4 patients with Ta stage (3.36%), 65 with T1 stage (54.62%), 22 with T2 stage (18.49%), 18 with T3 stage (15.13%), and 10 with T4 stage (8.40%). There was a total of 75 patients with high-grade urothelial carcinoma (63.03%) and 44 patients with low-grade urothelial carcinoma (36.97%). In the test set, there were 30 patients, including 23 males (76.67%) and 7 females (23.33%), with a mean age of 65.16 ± 9.43 years. The distribution of Ta, T1, T2, T3, T4 stages was 1 (3.33%), 5 (16.67%), 8 (26.67%), 11 (36.66%), 5 (16.67%) respectively, with 13 (43.33%) cases of low-grade urothelial carcinoma and 17 (56.67%) cases of high-grade urothelial carcinoma.

**Table 1:** Patient cohort.

	Training cohort (n = 119)	Test cohort (n = 30)
male (n, %)	101 (84.87%)	23 (76.67%)
female (n, %)	18 (15.13%)	7 (23.33%)
age (years, mean ± SD)	66.52 ± 12.62	65.16 ± 9.43
Tumor stage		
Ta	4 (3.36%)	1 (3.33%)
T1	65 (54.62%)	5 (16.67%)
T2	22 (18.49%)	8 (26.67%)
T3	18 (15.13%)	11 (36.66%)
T4	10 (8.40%)	5 (16.67%)
Pathological grade		
low-grade	44 (36.97%)	13 (43.33%)
high-grade	75 (63.03%)	17 (56.67%)

Basic information of patient.

### 3.2 Result of FISH assay

**Figure 1:**
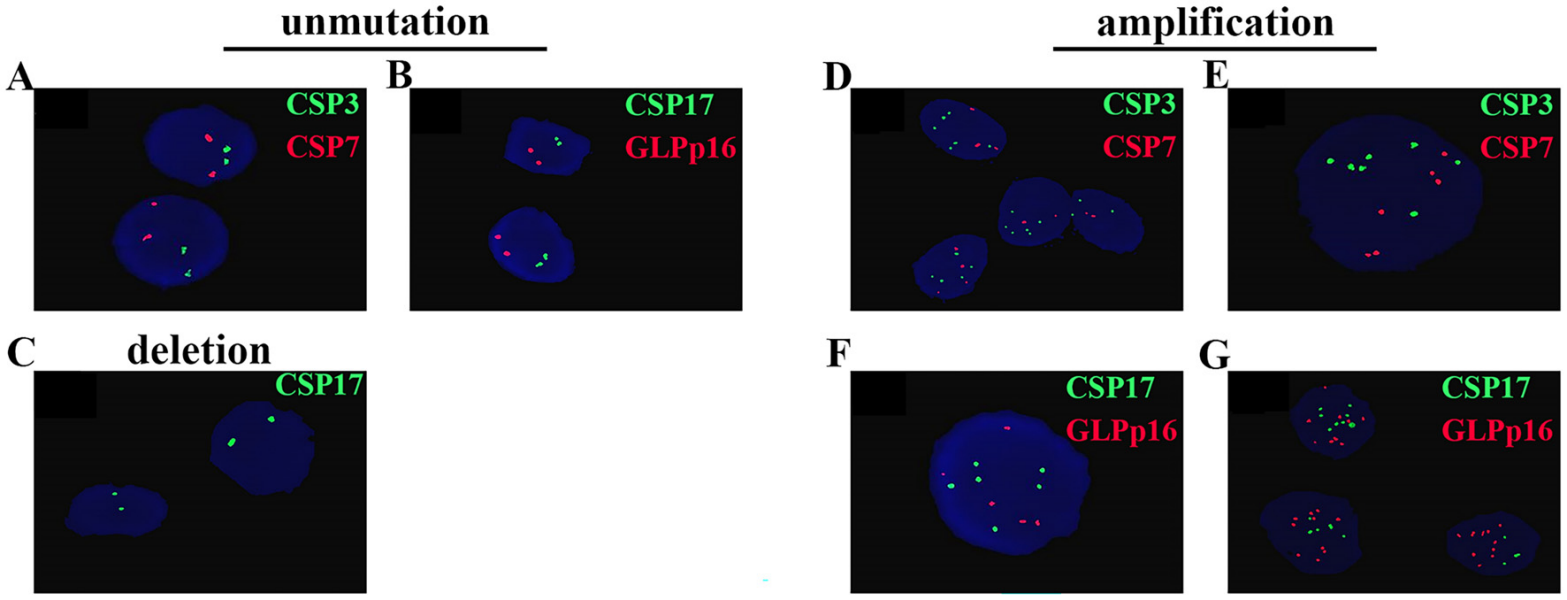
Cells without mutations exhibit two green fluorescent signals and two red fluorescent signals **(A, B)**. **(C)** Shows the deletion of GLPp16. **(D-G)** Sequentially display the amplifications of CSP3, CSP7, CSP17, and GLPp16.

**Figure 2:**
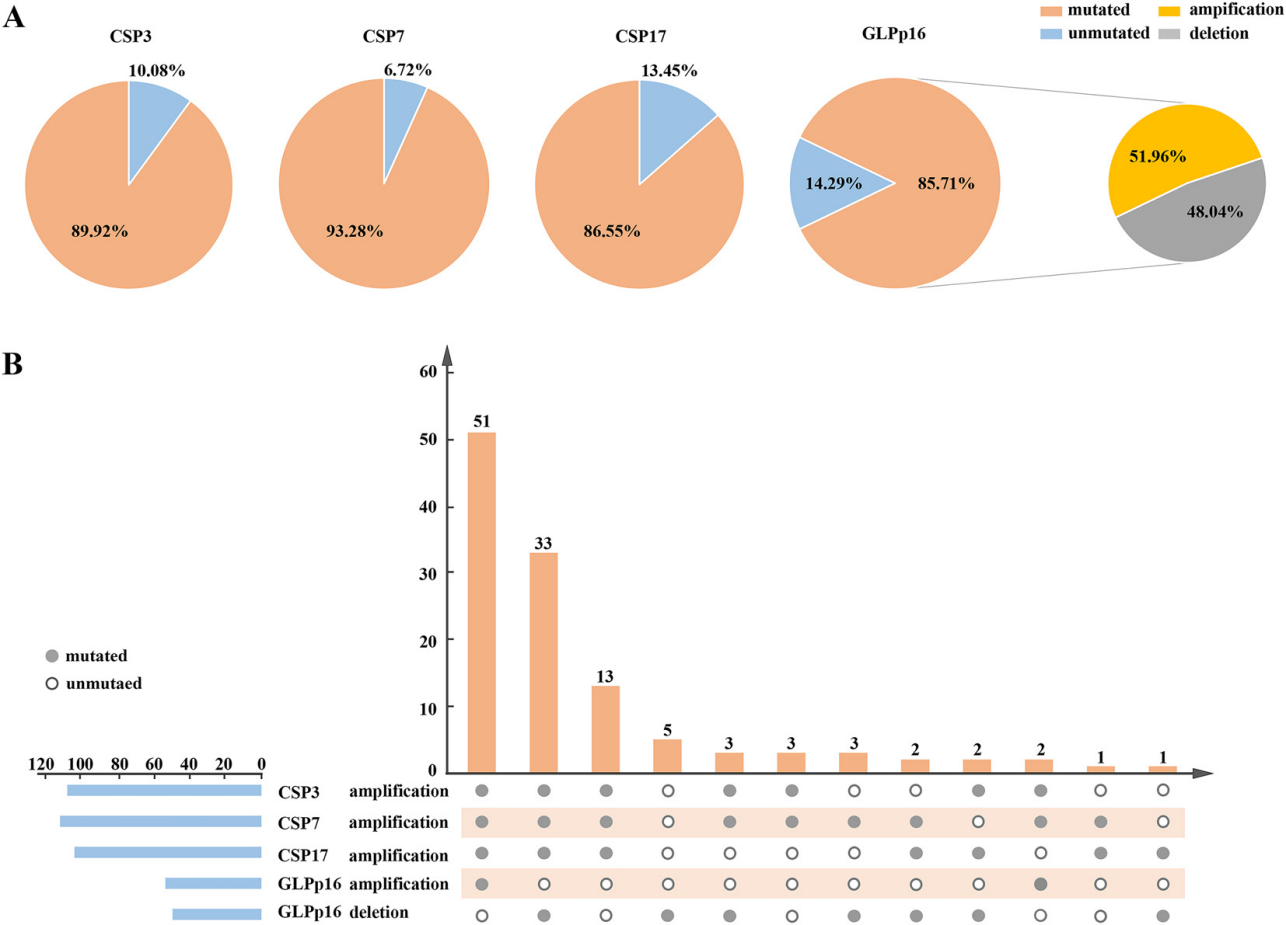
**(A)** A pie chart illustrating the distribution of FISH detection results for all patients in the training set was presented, depicting the state distribution of four FISH probes. **(B)** It described a flip chart showing the distribution of various types of positive FISH probe combinations. The horizontal axis used connected points to indicate different types of positive FISH probe combinations, while the vertical axis presented the patient count for specific combinations. The horizontal blue bars represented the positive counts for each FISH probe. FISH, Fluorescence *In Situ* Hybridization; CSP, Chromosome-specific centromere probe; GLP, Gene locus-specific probe.

The results of FISH depicted in **Figure 1** revealed diploid normal cells displaying 2 red/2 green fluorescent signals under the microscope when labeled with the probe. The CAS3 and CSP7 probes, respectively targeting chromosome 3 and chromosome 7, exhibited green and red fluorescence. Similarly, the CSP17 and GLPp16 probes, labeling the centromere of chromosome 17 and the p21 locus of chromosome 9, displayed green and red fluorescence, respectively. Anomalies in the deletion or amplification of red or green fluorescent signals detected by fluorescence signal implied abnormalities, signifying chromosomal deletions or amplifications. Notably, mutations were identified in chromosomes 3, 7, and 17, indicating amplifications, while chromosome 9 p21 site mutations involved both amplifications and deletions. Research data indicated that 89.92%, 93.28%, and 86.55% of patients respectively exhibited amplification of chromosomes 3, 7, and 17, while 85.71% of patients displayed a mutation in chromosome 9p21. Specifically, among all patients with mutations in GLPp16, 51.96% showed amplification while 48.04% exhibited deletion. (**Figure 2A**). The study further delineated four chromosomal mutation combinations observed in the 119 patients, as represented in **Figure 2B**. Among these, CSP3, CSP7, CSP17, and GLPp16 mutations were amplified in 51 patients. Additionally, 33 patients demonstrated amplifications in CSP3, CSP7, and CSP17 along with GLPp16 deletions, while 13 patients displayed amplifications in CSP3, CSP7, and CSP17 alongside GLPp16 unmutations. Furthermore, five patients exclusively exhibited GLPp16 deletions, without mutations in CSP3, CSP7, and CSP17.

### 3.3 Univariate analysis

**Figure 3:**
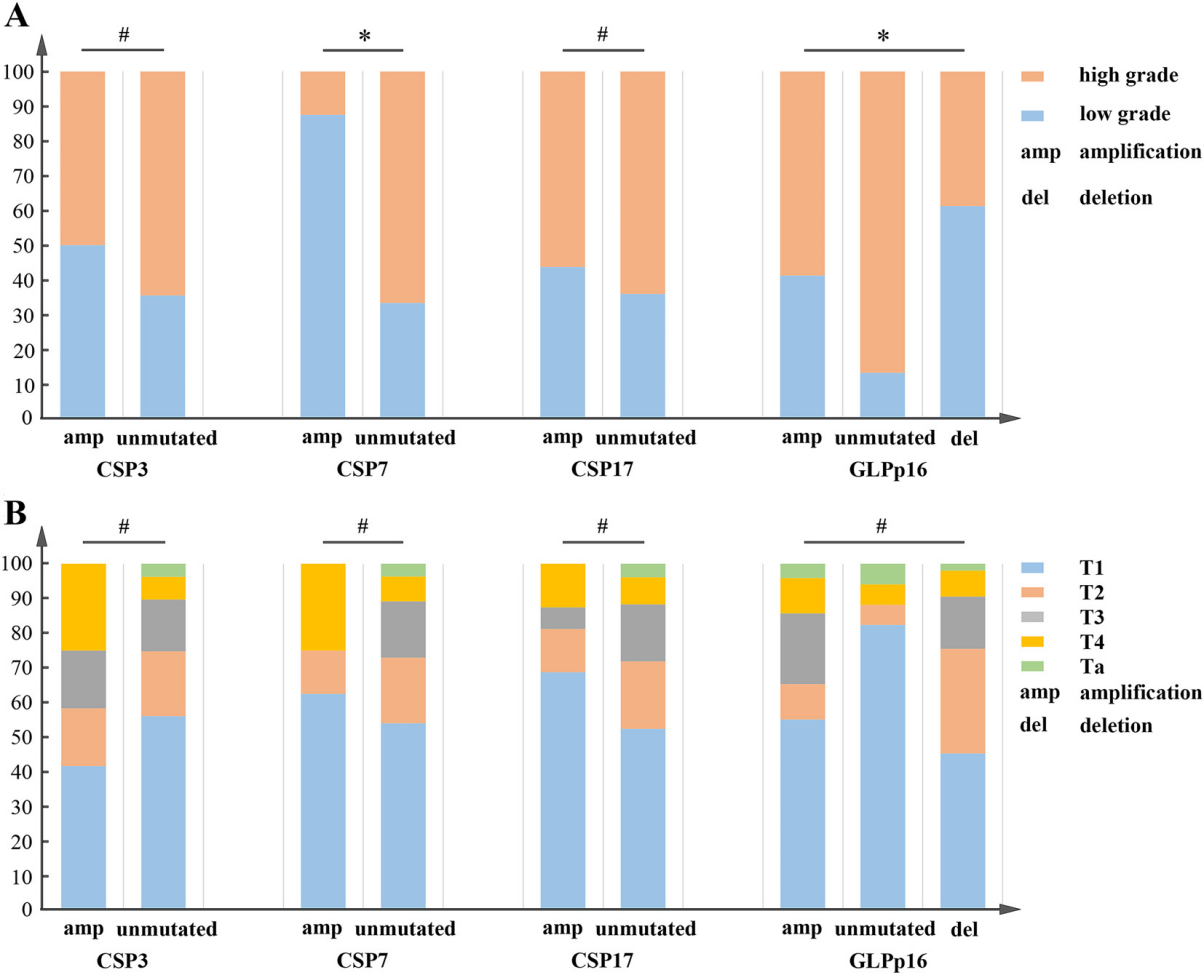
**(A)** CSP3 and CSP17 failed to show a significant correlation with the pathological grading of urothelial carcinoma, whereas CSP7 and GLPp16 were statistically associated with it. χ2(CSP3) = 0.97, χ2(CSP17) = 0.36, *p >* 0.05; χ2(CSP7) = 9.39, χ2(GLPp16) = 25.34, *p <* 0.05. **(B)** CSP3, CSP7, and GLPp16 showed no significant correlation with the TNM staging of urothelial carcinoma. χ2(CSP3) = 5.28, χ2(CSP7) = 4.66, χ2(CSP17) = 2.99, χ2 = 15.19, *p >* 0.05. *p < 0.05, #*p* > 0.05.

Our study findings, illustrated in **Figure 3A**, indicated a significant correlation between urothelial carcinoma pathological grade and the amplification of CSP7 as well as aneuploidy at GLPp16 (χ2 (CSP7) = 9.39, χ2 (GLPp16) = 25.34, *p <* 0.05) within the entire training cohort. Conversely, the amplification of CSP3 and CSP17 did not exhibit statistical variance between the study groups (*p >* 0.05). Furthermore, comparisons between aneuploidy alterations at CSP3, CSP7, CSP17, and GLPp16 did not reveal significant discrepancies with respect to TNM staging of urothelial carcinoma (**Figure 3B**, *p >* 0.05).

### 3.4 Establishment and verification of nomogram

**Figure 4:**
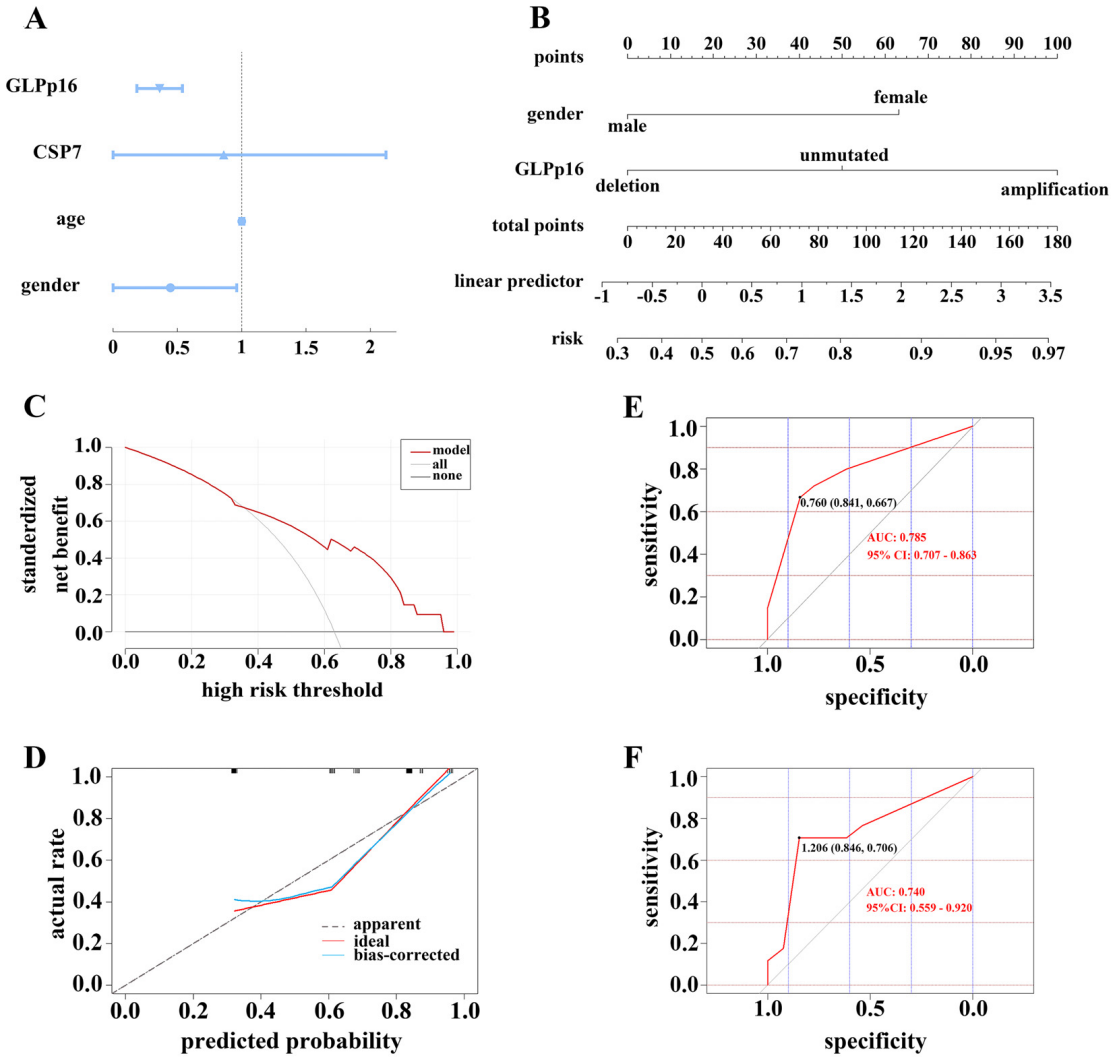
**(A)** Forest plot showing the correlation between FISH assay results and tumor grade. **(B)** A regression model was used to construct a FISH-clinical nomogram to predict the risk of high-grade pathological grading in patients with urothelial carcinoma. The risk score could be calculated based on the regression formula. **(C)** DCA of nomogram in training set. **(D)** Calibration curve of the cytogenetic-clinical nomogram in the training set, Hosmer-Lemeshow test: *p =* 0.436. Receiver operator characteristic curve of the cytogenetic-clinical nomogram in the training set **(E)** and validation set **(F)**.

In our investigation utilizing logistic regression analysis, we assessed the influence of gender, age, and aneuploidy changes in CSP7 and GLPp16 on the occurrence of low-grade urothelial carcinoma. The analysis revealed that gender and amplification of GLPp16 were inversely associated with the development of low-grade urothelial carcinoma (**Figure 4A**, *p <* 0.05), whereas age and amplification of CSP7 did not show a significant association with urothelial carcinoma stage (*p >* 0.05). Based on these findings, we constructed a nomogram model incorporating gender and GLPp16 (**Figure 4B**). The DCA results (**Figure 4C**) indicated that utilizing the nomogram model to make treatment decisions conferred greater net benefit across various threshold probabilities ranging from 36% to 95% compared to treating all patients or none. The calibration curve for the nomogram training set (**Figure 4D**) exhibited a non-significant Hosmer-Lemeshow test result (*p =* 0.436), signifying strong calibration ability and alignment between model predictions and actual observations. Furthermore, the ROC curve of the nomogram displayed an area under the curve (AUC) of 0.785 (95% CI: 0.707 - 0.863), demonstrating excellent discrimination (**Figure 4E**), while the ROC curve of the test set revealed an AUC of 0.740 (95% CI: 0.559 - 0.920), indicating robust discrimination capability in practical application (**Figure 4F**).

### 3.5 Immunohistochemistry

**Figure 5:**
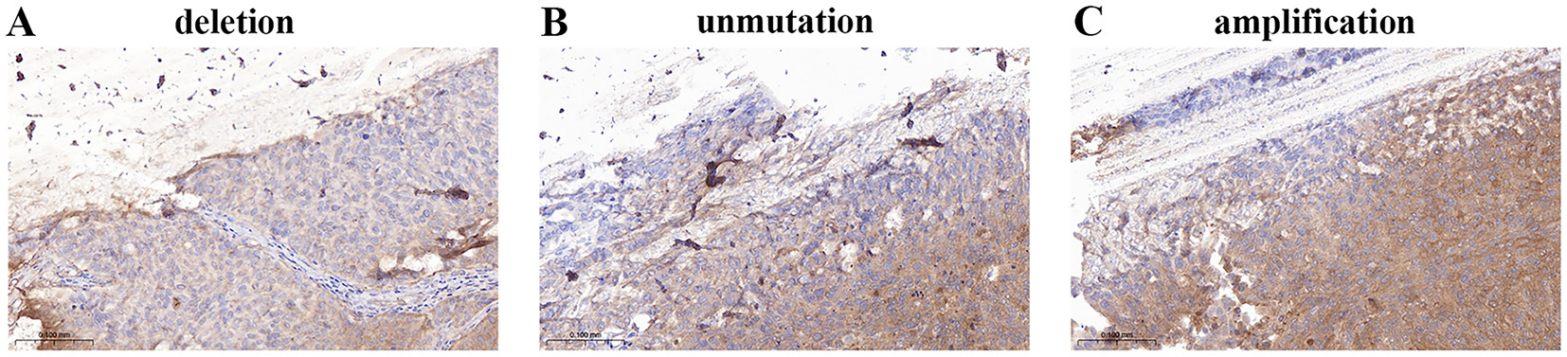
**(A)** Deletion of GLPp16 in low-grade urothelial carcinoma. **(B)** Unmutation of GLPp16 in high-grade urothelial carcinoma. **(C)** Amplification of GLPp16 in high-grade urothelial carcinoma.

The P16 locus on chromosome 9 tended to be deleted in low-grade urothelial carcinoma (**Figure 5A**) and amplified in high-grade urothelial carcinoma (**Figure 5B**). **Figure 5C** shows that GLPp16 was unmutated in high-grade urothelial carcinoma.

## 4 Discussion

Bladder cancer is the predominant urinary tract cancer globally, ranking as the 10th most common cancer (12). It disproportionately affects men, comprising 5% of male malignancies (1). In China, there were 85,000 new bladder cancer cases in 2020, representing 14.9% of global cases, with 39,000 deaths accounting for 18.5% worldwide (1, 13). Urothelial carcinoma is the most prevalent bladder cancer type, while squamous cell carcinoma, sarcoma, lymphoma, and adenocarcinoma are rarer (14, 15). It is noteworthy that upper tract urothelial carcinoma (UTUC) is less common than bladder cancer and has a poor prognosis. Its pathological grading and TNM staging are significant risk factors for postoperative recurrence (5, 13, 16). Therefore, timely detection, diagnosis, and treatment are crucial for reducing recurrence risk and alleviating patient burden. Although the incidence of UTUC is on the rise, only the American Urological Association (AUA), the European Association of Urology (EAU), and the National Comprehensive Cancer Network (NCCN) have released relevant guidelines. Research indicates that there is considerable variability in the recommendations and opinions within these guidelines, which may impact clinical practice (17). Thus, there is an urgent need for more high-quality studies to reach a consensus and enhance the diagnostic efficiency of urothelial carcinoma.

Currently, the gold standard for diagnosing urothelial carcinoma is pathologic examination of tumor tissue obtained through cystoscopy or ureteroscopy (4). However, these procedures are invasive, costly, and may not effectively detect early-stage tumors or *in situ* bladder cancer (18). While urinary cytology is non-invasive and convenient, its sensitivity ranges from 35% to 65% and lacks pathological grade information for effective treatment guidance (19). There is a pressing need for a more reliable, non-invasive, and cost-effective diagnostic method, emphasizing the importance of combining traditional and innovative testing technologies.

Research has shown that the occurrence of urothelial carcinoma is closely related to genetic alterations. For instance, patients with Lynch syndrome have a lifetime risk of developing UTUC of 9%, while the risk of bladder cancer is only 1%, representing a 22-fold increase compared to the general population (20). Furthermore, certain genes are associated with the staging and grading of urothelial carcinoma; among these, FGFR3 is the most commonly mutated gene in UTUC, with a mutation frequency as high as 74%, and this frequency can reach 92% in low-grade tumors (21). Therefore, the detection of genes associated with urothelial carcinoma has become an important method for diagnosing this disease. Common markers for urothelial carcinoma include FGFR3 and P53 genes. Although FGFR3 mutations are relatively common in low-grade bladder cancer, not all patients with urothelial carcinoma exhibit FGFR3 mutations. Thus, FGFR3 testing may lead to missed diagnoses in some cases, limiting its sensitivity (22). Furthermore, since P53 and FGFR3 mutations are not specific to urothelial carcinoma, similar mutations may also be found in other types of tumors or benign lesions, which reduces their specificity (22, 23). Therefore, there is an urgent need for a diagnostic method that possesses both high sensitivity and specificity. FISH accurately diagnoses tumor development by targeting genetic alterations. Widely used in urothelial carcinoma research, FISH has evolved into UroVysion FISH for liquid-based diagnostics, achieving a sensitivity of up to 87.8% and specificity of up to 85.7% (24, 25). Positive FISH results, alone or combined with traditional methods, enhance diagnostic efficiency for predicting recurrence, evaluating treatment efficacy, and monitoring follow-up (11, 24).

In urothelial carcinoma, chromosomal abnormalities in chromosomes 3, 7, and 17, along with variations in the P16 gene locus on chromosome 9, are closely associated with tumor initiation and progression (26). Studies have explored using fluorescence probes to target these chromosomes and gene locus for FISH detection in diagnosing urothelial carcinoma (26, 27). The identification of CSP/CSP7, CSP17/GLPp16 has emerged as critical for diagnosing urothelial carcinoma, aiding in determining muscle invasion and predicting recurrence (26, 27).

To investigate the role of FISH detection of CSP/CSP7, CSP17/GLPp16 in urothelial carcinoma diagnosis and patient prognosis, we analyzed chromosomal mutations, TNM staging, and pathological grades in urothelial carcinoma patients who underwent FISH testing between January 2022 and December 2023. Among all patients, there was a predominance of males, which is consistent with global trend. However, the average age of onset for these patients was 65-66 years, aligning with the average age in China but lower than that in Western populations (28, 29). This discrepancy may be attributed to racial differences and the unique etiological factor of aristolochic acid in the Chinese population (30). Furthermore, we observed that these patients exhibited lower tumor grades, but higher tumor stages compared to Western populations, which may also be related to the influence of aristolochic acid (30, 31).

The study revealed mutations in CAS3, CAS7, CSP17, and GLPp16 were prevalent in urothelial carcinoma patients, with amplified and deleted GLPp16 mutations observed. Chi-square testing indicated mutations in CAS3, CAS7, CSP17, and GLPp16 were not significantly correlated with TNM stage, while mutations in CSP7 and GLPp16 were significantly linked to pathological grade. Hence, we conducted a further investigation to determine if CSP7 and GLPp16 acted as independent factors impacting urothelial carcinoma grading. Moreover, it identified age 50-70 years and male gender as risk factors for urothelial cancer, and age was taken into consideration in our analysis. Therefore, we employed binary logistic regression analysis to investigate the impact of age, gender, CSP7, and GLPp16 on urothelial carcinoma pathological grade. The results revealed that only gender and GLPp16 were found to be independent risk factors for urothelial carcinoma grade. Consequently, a predictive model was devised to evaluate urothelial cancer grade based on gender and GLPp16 mutation status, showcased through a nomogram. The efficacy of the predictive model was evaluated through both internal and external validation processes, with the results confirming the model’s robust predictive performance.

Nonetheless, our study had limitations. Firstly, we had a limited sample size, resulting in less accurate predictive models. Secondly, patient survival and recurrence data were not gathered. For future research, we plan to enlarge the sample size and enhance the analysis of pertinent data to address these shortcomings.

## 5 Conclusion

Mutations at the GLPp16 were determined to be significantly correlated with the pathological grade of urothelial carcinoma. Additionally, the amplification of GLPp16 was recognized as a contributing factor to the development of high-grade urothelial carcinoma.

## Data Availability

The raw data supporting the conclusions of this article will be made available by the authors, without undue reservation.
